# Subjective Physical Symptoms Related to Bad Weather Among Persons Undergoing Medical Check-Up: A Single-Center Observational Study

**DOI:** 10.7759/cureus.50642

**Published:** 2023-12-16

**Authors:** Tetsuya Akaishi, Toshiaki Saito, Michiaki Abe, Tadashi Ishii

**Affiliations:** 1 Department of Education and Support for Regional Medicine, Tohoku University Hospital, Sendai, JPN; 2 Department of General Medicine, Kesennuma City Municipal Motoyoshi Hospital, Kesennuma, JPN

**Keywords:** physical symptoms, prevalence, irritability, headache, bad weather

## Abstract

Background and aim

The prevalence and characteristics of physical complaints related to bad weather among the general population remain poorly understood. This study aimed to elucidate the characteristics of subjective physical symptoms related to bad weather.

Methods

A cross-sectional survey was conducted by using self-reported health-related questionnaires obtained from individuals undergoing annual medical check-ups at a municipal hospital in Japan. Participants were asked about the presence and details of physical symptoms related to bad weather, together with other health-related questions.

Results

Among the 133 participants, 42 (32%) (95%CI 24-40) reported experiencing physical conditions related to bad weather. Among these 42 patients, the most common ailment was headache (67%; n=28), followed by low back pain (21%; n=9), fatigue (19%; n=8), and stiff neck/shoulder discomfort (12%; n=5). Comparison between individuals with and without bad weather-related conditions revealed that those affected were younger (p=0.0014) and exhibited higher numerical rating scale scores for gastrointestinal problems (p=0.0027), irritability/agitation (p<0.0001), and sleep disorders (p=0.0295). These associations were confirmed even after adjusting for age and sex.

Conclusions

Physical conditions related to bad weather, represented by headache, fatigue, and back pain, can be seen in 25-40% of the general population, especially in younger age groups. Individuals with these conditions are more likely to experience irritability/agitation, gastrointestinal problems, and sleep disorders.

## Introduction

Weather is known to trigger various physical symptoms and mental disturbances in the general population [1,2]. The common weather-related parameters that potentially trigger health problems include temperature and humidity. Common complaints associated with weather include joint pain, headache, cough, back pain, fatigue, and skin problems [1]. A previous headache diary-based study from Taiwan revealed the possible association between the perception of temperature sensitivity and headache incidence among patients with migraine [3]. Another headache diary-based study from Japan revealed an association between low barometric pressure and migraine attack frequency [4]. A study from Norway revealed that low barometric pressure increased pain and stress levels in patients with fibromyalgia [5]. Other studies also suggested an association between weather conditions and osteoarthritis pain [6-9]. Meanwhile, another study from Australia demonstrated no association between weather parameters, including barometric pressure, and the risk of a low back pain episode [10]. A systematic review also found inconsistency among the evaluated studies regarding the association between weather factors and pain level in people with rheumatoid arthritis, although the authors did not exclude the possibility that some patients with the disease may react differently to the weather [11]. Currently, data regarding the relationship between bad weather and subjective physical complaints among the general population are missing. Moreover, the detailed profiles of the physical complaints related to bad weather remain unknown. Therefore, the present study aimed to clarify the subjective physical symptoms related to bad weather in the general population through a self-reported questionnaire study regarding bad weather-related physical conditions among residents living in a city in Japan.

## Materials and methods

Study design

This study was conducted at Kesennuma City Municipal Motoyoshi Hospital in Japan from April 2020 to May 2023. The facility is the only hospital in the Motoyoshi District, home to approximately 10,000 residents. Paper-based self-reported questionnaire regarding health and lifestyle was used. Data were collected from adult residents of the city, who visited the hospital to undergo an annual medical check-up. Participants were recruited irrespective of the presence of past medical histories.

Climate of the study area

Kesennuma City is located in the Pacific coast region in the Tohoku district (northeastern Japan). The climate of the city is between temperate and continental climates. With the Köppen climate classification, the city belongs to the "Cfa" (Temperate - No dry season - Hot summer) areas, but the city is also close to "Dfa" (Continental - No dry season - Hot summer) area. Most areas of the main island of Japan belong to the Köppen's "Cfa" classification; therefore, the climate of Kesennuma City can be regarded as roughly the same as other areas of the country. Based on the data released from the Japan Meteorological Agency (https://www.jma.go.jp/jma/indexe.html), the annual average temperature of Kesennuma City is approximately 11℃, which peaks in August. The annual total rainfall is 1300-1400 mm, peaking between July and September. The monthly rainfall is over 30 mm in all months throughout the year.

Collected variables

The presence and details of physical symptoms, possibly attributable to bad weather, were self-reported by the participants as the main focus of this study. The following additional data were further collected as potential confounding factors: (i) demographic data: age and sex; (ii) comorbidities: past histories hypertension, diabetes mellitus, dyslipidemia, hyperuricemia, liver dysfunctions, malignancies, obesity, herpes zoster, herpes labialis, and periodontal disease; (iii) lifestyle: average length of sleep (hours), wake-up time on weekdays, alcohol consumption, tobacco use, mouth breathing, and exercise habits.

Furthermore, to search for unrecognized symptoms potentially related to bad weather, the following specific symptoms that are commonly observed were evaluated: unexplained complaints with psychosomatic disorders or fibromyalgia, such as fatiguability, gastrointestinal problems, irritability/agitation, cold intolerance, edema, sleep disorders [12,13]. The level of each specific symptom in the past month was measured by the Numerical Rating Scale (NRS) between 0 and 10.

Statistical analysis

Distributions of the continuous variables were described as the median and interquartile range (IQR), 25-75 percentiles. Comparisons of continuous variables between two groups were performed by the Mann-Whitney U test, and comparisons of frequencies were performed by the chi-square test with Yates continuity correction or Fisher’s exact test according to the number of patients in each subgroup. Multivariable analyses to evaluate the association between each of the self-reported systemic conditions and the presence of weather-related symptoms were performed by the multiple linear regression analysis, adjusting for age and sex. P-values less than 0.05 were considered to be statistically significant, and adjustment for the multiple comparisons was not performed. All statistical analyses were performed with the R Statistical Software (R Foundation, Vienna, Austria).

Ethics

This study was approved by the Institutional Review Board of Tohoku University Hospital (approval number: 20201063). Written informed consent was obtained from all participants. All the study processes were performed in accordance with the latest version of the Declaration of Helsinki as revised in 2013.

## Results

Study population

A total of 151 adults (77 males and 74 females) agreed to answer the questionnaires and returned the sheets. Among them, 133 adults (71 males and 62 females) answered the question regarding the presence of any subjective physical conditions related to bad weather, such as rainy or snowy days. The median (IQR) age of the 133 individuals was 42 (31-56) years. This age distribution was lower than the median age in Kesennuma City (median: 55-60 years).

Physical conditions related to bad weather

**Figure 1:**
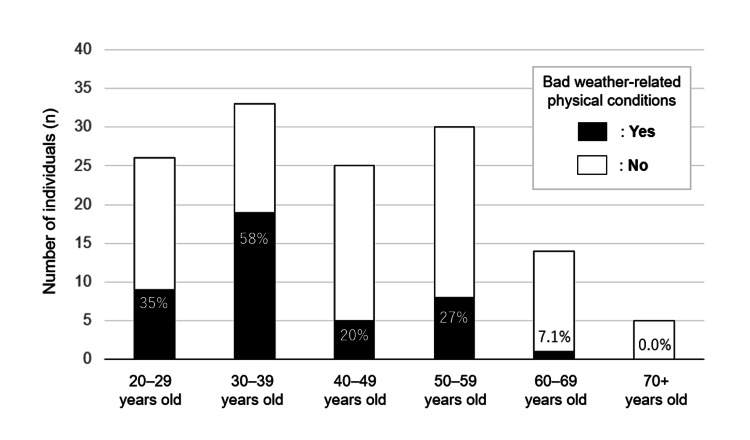
Prevalence of bad weather-related physical conditions by age groups The prevalence of self-reported bad weather-related physical conditions was the highest in the age group of 30–39 years (58%), followed by 20–29 years (35%). The prevalence was less than 10% in older populations aged ≥60 years.

Among the 133 participants who completed the questionnaire, 42 (24 males and 18 females) reported having any kind of subjective physical conditions related to bad weather, whilst the remaining 91 (47 males and 44 females) did not. The estimated prevalence (95%CI) of the conditions in the community population was 32% (95%CI 24-40). The prevalence of the conditions by age group is shown in Figure 1. The prevalence was the highest in the age group of 30-39 years (58%), followed by 20-29 years (35%). All 42 individuals with the conditions offered the details of their conditions. The reported bad weather-related physical conditions, allowing overlap, are summarized in Table 1. The most common condition was headache (67%), followed by low back pain (21%), fatigue (19%), and stiff neck/shoulder (12%).

**Table 1:** List and demographics of bad weather-related subjective physical conditions The list of self-reported bad weather-related conditions, allowing overlap, is shown in the order of the prevalence. ^*^Age distribution of the conditions with three or less individuals was shown by listing all ages. IQR: interquartile range

Conditions	Prevalence, n (%)	Male, n (%)	Age, median (IQR) *
Headache	28/42 (67%)	12/28 (43%)	33.5 (28–36) years
Low back pain	9/42 (21%)	7/9 (78%)	35 (33–36) years
Fatigue	8/42 (19%)	4/8 (50%)	57 (24–59) years
Stiff neck/shoulder	5/42 (12%)	1/5 (20%)	28 (27–32) years
Wheezing	3/42 (7.1%)	3/3 (100%)	40, 52, 53 years
Arthralgia (joint pain)	3/42 (7.1%)	2/3 (67%)	25, 52, 67 years
Dizziness/light-headedness	2/42 (4.8%)	1/2 (50%)	30, 32 years
Tinnitus	2/42 (4.8%)	1/2 (50%)	35 years for both
Pain from old wounds	1/42 (2.4%)	0/1 (0%)	36 years

Backgrounds of individuals with bad weather-related physical conditions

**Figure 2:**
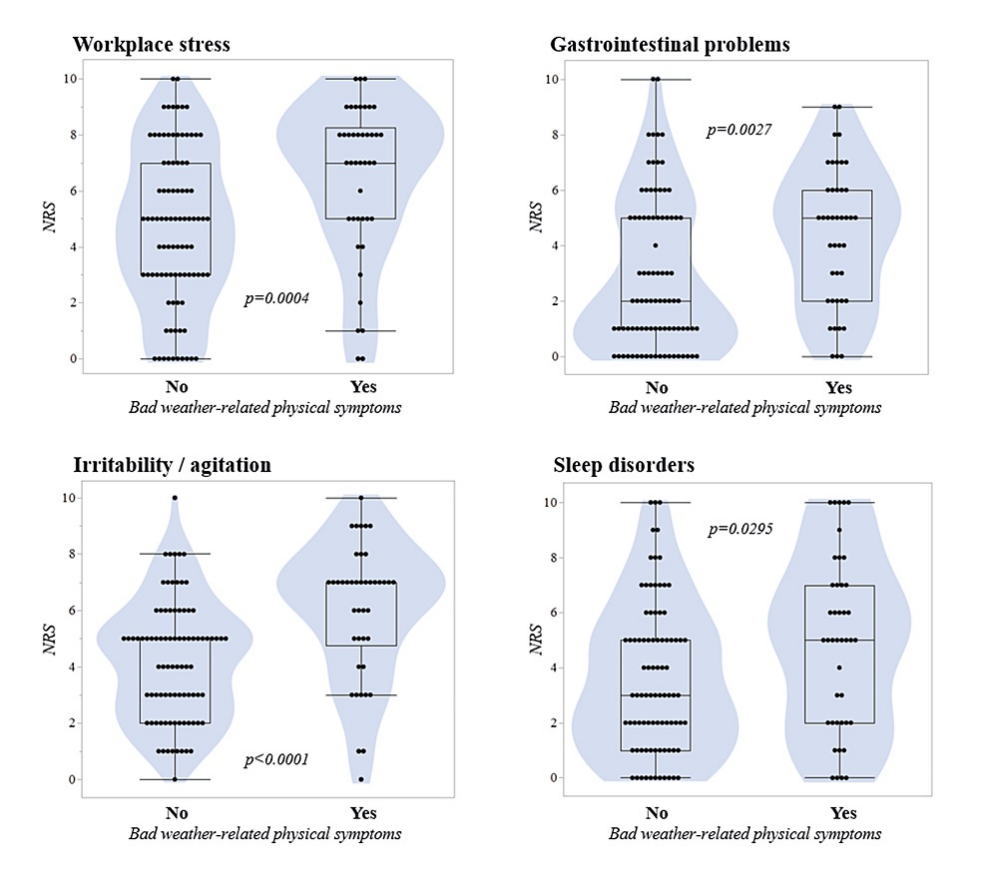
Violin plots of the NRS scores by the presence of bad weather-related physical symptoms The NRS scores for the recent workplace stress (p=0.0004), gastrointestinal problems (p=0.0027), irritability/agitation (p<0.0001), and sleep disorders (p=0.0295) in the last one month were significantly higher among those with self-reported bad weather-related physical symptoms compared to the others. NRS, Numerical Rating Scale

The demographic, lifestyle-related, and clinical backgrounds were compared according to the presence of bad weather-related physical conditions (Table 2). Individuals with the conditions were significantly younger (p=0.0014, Mann-Whitney U test). The male-to-female ratio did not differ between the groups (p=0.69). Any of the evaluated lifestyle-related backgrounds or the prevalence of past medical histories did not differ between the groups (p≥0.10 for all). Regarding the recent systemic conditions in NRS score, workplace stress (p=0.0004), gastrointestinal dysfunction (p=0.0027), irritability/agitation (p<0.0001), and sleep problems (p=0.0295) showed higher scores in those with bad weather-related conditions than in those without them. The violin plots for each of these four recent systemic conditions are shown in Figure 2.

**Table 2:** Demographic, lifestyle-related, and clinical data by the presence of bad weather-related physical conditions Distributions of continuous variables are described as the median and interquartile range (25–75 percentiles) unless otherwise mentioned

Characteristics	Weather-related physical conditions		P-value
	Yes (n=42)	No (n=91)	
Age	34 (30–44) years	47 (34.5–58) years	0.0014
Male, n (%)	24/42 (57%)	47/91 (52%)	0.6866
Chalder Fatigue Score (4–56)	34 (29–38)	31 (26–37)	0.0323
Lifestyle-related information			
Daily length of sleep	6.5 (6–7) hours	6.5 (6–7) hours	0.3269
Wake-up time	06:00 (05:20–06:30)	06:00 (05:30–06:30)	0.8310
Weekly alcohol consumption	0.0 (0.0 – 35) g	5.0 (0.0 – 45.5) g	0.5097
Daily numbers of tobacco use	10 (0–20)	10 (0–20)	0.7745
Mouth breathing	7/40 (18%)	22/84 (26%)	0.3999
Weekly exercise habit	0 (0–1) days	0 (0–1) days	0.7276
Past medical histories, n (%)			
Hypertension	5/42 (12%)	14/90 (16%)	0.7715
Diabetes mellitus	1/42 (2.4%)	7/90 (7.8%)	0.4349
Dyslipidemia	5/42 (12%)	19/90 (21%)	0.3006
Hyperuricemia	3/42 (7.1%)	3/90 (3.3%)	0.3820
Liver dysfunction	5/42 (12%)	4/90 (4.4%)	0.1425
Malignancy	0/42 (0.0%)	4/90 (4.4%)	0.3062
Obesity	4/42 (9.5%)	6/90 (6.7%)	0.7251
Herpes zoster	3/41 (7.3%)	15/88 (17%)	0.2255
Herpes labialis	5/42 (12%)	17/89 (19%)	0.4366
Periodontal disease	16/32 (50%)	31/72 (43%)	0.6575
Symptoms in the last one month with Numerical Rating Scale (0–10)			
Family stress	5.0 (1.0–7.0)	2.5 (1.0–5.0)	0.0577
Workplace stress	7.0 (5.0–8.0)	5.0 (3.0–7.0)	0.0004
Fatigability	5.0 (4.0–7.0)	5.0 (3.0–6.0)	0.0719
Gastrointestinal problems	5.0 (2.0–6.0)	2.0 (1.0–5.0)	0.0027
Irritability/agitation	7.0 (5.0–7.0)	5.0 (2.5–5.0)	<0.0001
Cold intolerance	5.0 (3.0–5.0)	5.0 (5.0–5.0)	0.1444
Edema	2.0 (0.5–6.0)	1.5 (0.0–3.0)	0.0554
Sleep disorders	5.0 (2.0–7.0)	3.0 (1.5–5.0)	0.0295

Multiple linear regression analysis for each systemic condition

Finally, to evaluate the association between each evaluated systemic condition level and the presence of bad weather-related subjective physical conditions, a multiple linear regression analysis adjusting for age and sex was further performed for each of the additional conditions with p-values <0.05 in the univariate analyses (Table 3). All of the four additional conditions showed a statistically significant association with the presence of bad weather-related physical conditions, with the highest coefficient with irritability/agitation (=0.3557, p<0.0001). As sensitivity analyses, the same regression models were regenerated using the presence of bad weather-related headaches (Yes/No), instead of the presence of the overall bad weather-related physical conditions (Table 4). Statistically significant association with the presence of bad weather-related headaches was observed in the levels of workplace stress (=0.1982, p=0.0352), gastrointestinal problems (=0.2076, p=0.0300), and irritability/agitation (=0.3031, p=0.0013).

**Table 3:** Multiple linear regression analysis of the presence of bad weather-related symptoms for the level of each additional condition For each of the four regression models, one dependent variable (workplace stress, gastrointestinal problems, irritability/agitation, or sleep disorders) and three explanatory variables (age, sex, and the presence of bad weather-related symptoms) were used. Data of the bad weather-related symptoms was used as a binary data based on the presence of at least one self-reported condition. * Dummy variable (Male=1, Female=0; bad weather-related physical conditions “yes” = 1, “no” = 0) VIF, variance inflation factor; NRS, Numerical Rating Scale

Characteristics	Unstandardized coefficient		Standardized coefficient (β)	t	P-value	VIF
	B	Std. error				
Dependent variable: Workplace stress (NRS 0–10)						
Age	-0.0052	0.0176	-0.0264	-0.30	0.7676	1.123
Male *	0.1020	0.2477	0.0352	0.41	0.6812	1.031
Weather-related symptoms *	0.8821	0.2736	0.2834	3.22	0.0016	1.092
Dependent variable: Gastrointestinal problems (NRS 0–10)						
Age	–0.0000	0.0171	–0.0001	–0.00	0.9991	1.102
Male *	–0.0663	0.2383	–0.0243	–0.28	0.7814	1.021
Weather-related symptoms *	0.7357	0.2607	0.2538	2.82	0.0056	1.081
Dependent variable: Irritability/agitation (NRS 0–10)						
Age	–0.0040	0.0142	–0.0247	–0.28	0.7786	1.102
Male *	0.0111	0.1971	0.0047	0.06	0.9553	1.021
Weather-related symptoms *	0.8853	0.2156	0.3557	4.11	<0.0001	1.081
Dependent variable: Sleep disorders (NRS 0–10)						
Age	–0.0013	0.0185	–0.0063	–0.07	0.9457	1.102
Male *	0.0126	0.2569	0.0044	0.05	0.9609	1.021
Weather-related symptoms *	0.6188	0.2810	0.2005	2.20	0.0295	1.081

**Table 4:** Multiple linear regression analysis of the presence of bad weather-related headaches for the level of each additional condition For each of the four regression models, one dependent variable (workplace stress, gastrointestinal problems, irritability/agitation, or sleep disorders) and three explanatory variables (age, sex, and the presence of bad weather-related headaches) were used. * Dummy variable (Male=1, Female=0; bad weather-related headaches “yes” = 1, “no” = 0) VIF, variance inflation factor; NRS, Numerical Rating Scale

Characteristics	Unstandardized coefficient		Standardized coefficient (β)	t	P-value	VIF
	B	Std. error				
Dependent variable: Workplace stress (NRS 0–10)						
Age	–0.0067	0.0186	-0.0342	-0.36	0.7170	1.198
Male *	–0.2028	0.2574	–0.0699	–0.79	0.4323	1.067
Weather-related headaches *	1.407	0.6611	0.1982	2.13	0.0352	1.175
Dependent variable: Gastrointestinal problems (NRS 0–10)						
Age	0.0011	0.0179	0.0056	0.06	0.9527	1.174
Male *	–0.0320	0.2453	–0.0117	–0.13	0.8964	1.056
Weather-related headaches *	1.368	0.6231	0.2076	2.20	0.0300	1.166
Dependent variable: Irritability/agitation (NRS 0–10)						
Age	–0.0020	0.0149	–0.0122	–0.13	0.8950	1.174
Male *	–0.1343	0.2048	–0.0573	–0.66	0.5134	1.056
Weather-related headaches *	1.715	0.5203	0.3031	3.30	0.0013	1.166
Dependent variable: Sleep disorders (NRS 0–10)						
Age	–0.0073	0.0194	–0.0364	–0.38	0.7070	1.174
Male *	–0.0474	0.2657	–0.0163	–0.18	0.8588	1.056
Weather-related headaches *	0.4793	0.6750	0.0683	0.71	0.4790	1.166

## Discussion

In this study, self-reported physical symptoms related to bad weather (i.e., rainy or snowy days) were evaluated among the general population living in a local city in Japan. The obtained results suggested that 24-40% of the general population may experience physical symptoms/conditions on days with such weather conditions. The most common physical symptom was headache, followed by back pain and fatigue. The presence of bad weather-related conditions was associated with various physical and mental conditions, such as irritability/agitation, workplace stress, fatigability, gastrointestinal symptoms, and sleep disorders, suggesting that some of these conditions may be incorporated into the spectrum of weather-related symptoms.

Association between gastrointestinal problems and weather factors has not been reported in the general population till now, but a recent meta-analysis suggested the presence of seasonal variation in bowel symptoms among patients with inflammatory bowel disease [14]. Another recent study from Japan showed that the incidence of unexplained adhesive small bowel obstruction is susceptible to barometric pressure and humidity [15]. The authors hypothesized the presence of visceral hypersensitivity against changes in barometric pressure and air temperature. A case-control study from Japan investigating patients with ischemic colitis found that low or decreased barometric pressure is associated with the onset of ischemic colitis [16]. Another study from Germany found an association between meteorological factors and variceal hemorrhage in the gastrointestinal tract [17]. These previous studies suggest the presence of visceral hypersensitivity against atmospheric pressure and/or temperature.

An association between sleep disturbance and bad weather has been also suggested earlier. In a previous study with patients suffering from sleep disorders, the obstructive apnea index was found to increase with lower barometric pressure [18]. The present study also suggested the possibility that pains related to bad weather may be other conceivable mechanisms underlying sleep disorders among patients with weather-related physical conditions. Further studies are needed to elucidate the mechanisms connecting low barometric pressure and these conditions potentially related to low barometric pressure.

This study has several limitations. First, the number of participants was relatively small. As a result, false negative results could be possible in some evaluated characteristics. Second, this surveillance almost exclusively recruited people with Asian ancestry. Therefore, the generalizability of the findings to people of different races and ethnicities is unknown. Finally, the evaluated bad weather-related physical symptoms were subjective and were empirically attributed to bad weather by the participants themselves. The exact causal relationship between bad weather and each reported physical symptom remains uncertain. Nevertheless, the findings implied that bad weather-related symptoms may extend beyond the traditionally studied conditions and have broader implications for mental and physical health. Future studies with longitudinal follow-ups of daily symptoms and weather factors are needed to conclude the causation between them.

## Conclusions

Physical conditions related to bad weather, represented by headaches, back pain, fatigue, and joint pain, may be seen in 25-40% of the general population. The prevalence of bad weather-related physical conditions is higher in younger people, peaking at 30-39 years of age. The presence of these conditions is primarily associated with workplace stress and the co-existence of irritability/agitation, gastrointestinal problems, and sleep disorders. Establishing criteria for managing people with bad weather-related physical conditions is needed.
